# Intra-articular Platelet-Rich Plasma Injection for Pain and Functional Outcomes in Knee Osteoarthritis: A Prospective Cohort Study with Short-Term Follow-Up

**DOI:** 10.7759/cureus.117886

**Published:** 2026-10-13

**Authors:** Rumeshkannan Gunasekaran, Adimoolam Manikandan, Mohammed Noorul Ameen

**Affiliations:** 1 Department of Orthopaedics, Indira Gandhi Medical College and Research Institute, Puducherry, IND; 2 Department of Orthopaedics, Sri Venkateshwaraa Medical College Hospital and Research Centre, Puducherry, IND

**Keywords:** functional outcome, intra-articular injection, knee osteoarthritis, pain, platelet-rich plasma, womac

## Abstract

Background: Osteoarthritis (OA) is a chronic degenerative joint disorder associated with substantial morbidity and limited effective treatment options. Intra-articular platelet-rich plasma (PRP) injection has emerged as a potential therapeutic modality; however, clinical outcomes remain variable, necessitating further evaluation. This study aimed to evaluate changes in pain and functional outcomes following a single intra-articular PRP injection in patients with knee OA.

Methodology: This prospective cohort study evaluated the short-term outcomes of PRP injection in patients with knee OA. A total of 72 patients received a single intra-articular injection of 8 mL of PRP prepared from 60 mL of autologous venous blood using a double-centrifugation technique. Patients were assessed using the Western Ontario and McMaster Universities Osteoarthritis Index (WOMAC), Visual Analogue Scale (VAS) for pain, International Knee Documentation Committee (IKDC) subjective knee score, and six-minute walking distance (6MWD) at baseline and at one, three, and six months following injection.

Results: Statistically significant improvements were observed in WOMAC pain, stiffness, and physical function scores at one, three, and six months compared with baseline (p < 0.001). VAS pain scores decreased, whereas IKDC scores and 6MWD improved throughout the follow-up period. However, the magnitude of improvement declined at six months compared with earlier follow-up assessments.

Conclusion: Intra-articular PRP injection was associated with short-term improvements in pain and functional outcomes among patients with knee OA. However, given the absence of a control group and the limited follow-up duration, these findings should be interpreted with caution and do not establish a causal treatment effect. Further comparative studies with longer follow-up and standardized PRP characterization are warranted.

## Introduction

Osteoarthritis (OA) is a chronic degenerative joint disorder and a leading cause of long-term morbidity, characterized by pain, stiffness, and progressive functional limitation [1]. The knee is one of the most commonly affected joints, and established risk factors include advancing age, female sex, obesity, prior trauma, and muscle weakness [2]. The global burden of knee OA continues to increase, substantially affecting quality of life and placing considerable strain on healthcare systems [1].

Management of knee OA remains largely symptomatic and involves a combination of non-pharmacological, pharmacological, and surgical interventions [3,4]. Although total knee arthroplasty remains the definitive treatment for advanced disease, many patients seek less invasive therapeutic options to delay or avoid surgery.

In recent years, intra-articular platelet-rich plasma (PRP) injection has emerged as a potential biological therapy aimed at modulating inflammation and promoting tissue repair. Several clinical studies have reported improvements in pain and functional outcomes following PRP administration during short- to mid-term follow-up [5,6]. However, the available evidence remains heterogeneous, with conflicting findings reported across studies [7]. Systematic reviews and meta-analyses have suggested potential benefits of PRP; nevertheless, variability in study design, patient selection, and PRP preparation techniques limits the generalizability of these findings [8-11]. In particular, differences in platelet concentration, leukocyte content, and processing protocols have been proposed as important factors contributing to inconsistent clinical outcomes [12,13].

At our tertiary care center, intra-articular PRP injections are routinely used in the management of patients with symptomatic knee OA despite ongoing variability in the published evidence. However, treatment outcomes in our setting have not been systematically evaluated, highlighting the need for further investigation.

The primary objective of this study was to evaluate changes in Western Ontario and McMaster Universities Osteoarthritis Index (WOMAC) pain, stiffness, and physical function scores at one, three, and six months following a single intra-articular PRP injection. Secondary objectives were to assess changes in Visual Analog Scale (VAS) pain scores, International Knee Documentation Committee (IKDC) subjective knee scores, and six-minute walking distance (6MWD) over the same follow-up period.

## Materials and methods

Study design and setting

This prospective cohort study was conducted at a tertiary care teaching hospital in South India over one year, from November 2022 to October 2023. The study protocol was approved by the Institutional Ethics Committee, and all procedures were conducted in accordance with the principles of the Declaration of Helsinki. Written informed consent was obtained from all participants before enrollment.

Study population

Consecutive patients with symptomatic knee OA were included in the study. The diagnosis was established based on clinical assessment and radiographic findings using the Kellgren-Lawrence grading system [14], along with symptom severity assessed using the Western Ontario and McMaster Universities Osteoarthritis Index (WOMAC®) [15]. The WOMAC questionnaire was administered in its validated form, and the pain, stiffness, and physical function subscales were calculated separately according to their respective scoring methods. Higher scores indicated greater symptom severity, with lower scores representing improvement. The WOMAC pain, stiffness, and physical-function subscales had ranges of 0-20, 0-8, and 0-68, respectively.

Patients with a range of disease severity (Kellgren-Lawrence grades 2-4) were included to reflect the spectrum of disease encountered in routine clinical practice. Patients with secondary OA, active infection, serious systemic illness, recent intra-articular injections within the preceding six months, moderate-to-severe anemia, or thrombocytopenia were excluded.

Patients with symptomatic knee OA were included on a per-patient basis, with one affected knee designated as the study knee for treatment and outcome assessment. In patients with bilateral symptomatic knee OA, only one knee was selected for PRP treatment and designated as the study knee before the intervention. All clinical outcome assessments, including WOMAC, VAS, IKDC, and 6MWD, were performed with reference to the designated study knee throughout the follow-up period. The contralateral knee was neither treated nor included in the analysis.

Sample size

The sample size was calculated assuming that 30% of patients would experience significant pain relief at six months. Using a 95% confidence level and an absolute precision of 11%, the minimum required sample size was estimated at 69 participants. To account for potential loss to follow-up, the target sample size was increased to 80 participants. During the study period, 72 eligible participants were ultimately enrolled and received the intervention. These 72 participants were included in the final analysis.

Baseline assessment

Baseline demographic characteristics, comorbidities, and anthropometric measurements were recorded. Body mass index (BMI) was calculated and categorized according to World Health Organization (WHO) guidelines [16], which is a freely available public health classification system requiring no licensing or permission for academic use. Patients were advised to discontinue analgesic medications at least two weeks before the procedure.

Baseline outcome measures included the WOMAC® (University of Queensland, Brisbane, Australia) pain, stiffness, and physical function scores [15], the VAS for pain [17], the IKDC subjective knee score [18], and the 6MWD [19]. The VAS is a freely available and non-proprietary pain assessment tool widely used in clinical research with no licensing requirement. The IKDC subjective knee score is a validated patient-reported outcome measure freely available for academic and clinical research use with proper citation. The 6MWD is a standard functional capacity test developed under American Thoracic Society guidelines, which are publicly accessible and do not require licensing for use in research settings. All outcome measures were applied in their standard validated formats without modification.

PRP preparation and injection protocol

Approximately 60 mL of autologous venous blood was collected using acid citrate dextrose as an anticoagulant. PRP was prepared using a double-centrifugation technique designed to achieve a higher platelet concentration by separating red blood cells during the first centrifugation step and concentrating platelets during the second. The first centrifugation was performed at 1,700 rpm for 10 minutes, followed by a 10-minute resting period. The supernatant plasma layer containing platelets and leukocytes was transferred to a sterile tube and subjected to a second centrifugation at 2,500 rpm for 10 minutes. The supernatant platelet-poor plasma fraction was discarded, yielding approximately 10 mL of PRP. Subsequently, 8 mL of PRP was injected intra-articularly into the affected knee using a suprapatellar approach within one hour of preparation.

PRP was prepared using a double-centrifugation protocol as described above. Platelet concentration relative to baseline whole-blood platelet count was assessed in only three patients because routine cellular characterization was not available during the study period. Leukocyte concentration, platelet recovery, and activation status were not systematically measured. Centrifugation parameters were recorded in revolutions per minute (rpm); the rotor radius and corresponding relative centrifugal force (RCF) were not documented. Therefore, the exact cellular composition and quantitative platelet enrichment of the PRP preparation could not be determined for the study cohort. These factors have been recognized as limitations affecting the reproducibility and biological characterization of the intervention. Following injection, passive knee mobilization was performed, and patients were observed for any immediate adverse effects.

Follow-up and outcome measures

Patients were monitored for 24 hours following the procedure for the occurrence of immediate adverse events. Following PRP injection, patients were followed according to the study protocol at one, three, and six months. No additional PRP injection was planned during the six-month follow-up period. Concomitant interventions such as physiotherapy, structured exercise programs, weight-management interventions, brace use, and analgesic or NSAID use during follow-up were not systematically standardized across participants. Therefore, the potential influence of these factors on longitudinal changes in pain and functional outcomes cannot be entirely ruled out. Patients were instructed to report any adverse events or worsening symptoms, and telephone reminders were provided to enhance follow-up compliance. Outcome measures, including WOMAC® [15], VAS [17], IKDC [18], and 6MWD [19], were reassessed at each follow-up visit.

Statistical analysis

Data were entered into MS Excel (Microsoft Corporation, Redmond, Washington) and analyzed using Stata version 12 (StataCorp LLC, College Station, Texas). Baseline characteristics were summarized as frequencies and percentages for categorical variables and as mean ± standard deviation or median (interquartile range) for continuous variables, as appropriate based on data distribution. Comparisons of outcome measures across follow-up time points were performed using repeated-measures analysis of variance (ANOVA) for normally distributed data and the Friedman test for non-normally distributed data. The analysis was based on the repeated measurements obtained from the same participants over time. A two-sided p-value of <0.05 was considered statistically significant.

## Results

A total of 72 participants were included in the study, with a mean age of 57.1 ± 7.8 years. The majority of participants belonged to the 51-60-year age group, comprising 34 (47.2%) participants, and females accounted for 39 (54.2%) of the study population. The mean BMI was 18 ± 9.6 kg/m², and a normal BMI was reported in 28 (38.8%) participants. Diabetes mellitus was the most common comorbidity, affecting 22 (30.5%) participants, while manual labor was the predominant occupation, reported by 36 (50.0%) participants. Detailed baseline demographic and clinical characteristics are presented in Table 1.

**Table 1:** Baseline demographic and clinical characteristics of the participants (n = 72). BMI: body mass index.

Variable	Category	Value
Age (years)	Mean ± SD	57.1 ± 7.8
	41-50 years, n (%)	10 (13.9)
	51-60 years, n (%)	34 (47.2)
	61-69 years, n (%)	23 (31.9)
	≥70 years, n (%)	5 (6.9)
Gender	Male, n (%)	33 (45.8)
	Female, n (%)	39 (54.2)
Height (cm)	Mean ± SD	161.6 ± 7.2
Weight (kg)	Mean ± SD	60.4 ± 8.0
BMI	Mean ± SD	18 ± 9.6
	Underweight, n (%)	4 (5.5%)
	Normal weight, n (%)	28 (38.8%)
	Overweight, n (%)	26 (36.11%)
	Obese, n (%)	14 (19.4%)
Comorbidities	Hypertension, n (%)	11 (15.3)
	Diabetes mellitus, n (%)	15 (20.8)
	Diabetes mellitus + Hypertension, n (%)	7 (9.7)
	Thyroid disorders, n (%)	2 (2.8)
	Other comorbidities, n (%)	4 (5.6)
Nature of employment	Household activities, n (%)	15 (20.8)
	Manual labor, n (%)	36 (50.0)
	Minimal physical activity work, n (%)	21 (29.2)

At baseline, pain medication was used three times per week by 37 (51.4%) participants, and the mean duration of knee pain was 17.3 ± 7.0 months. A history of knee joint injury was reported in 16 (22.2%) participants. According to the Kellgren-Lawrence classification, Grade 3 OA was the most common radiographic finding, observed in 37 (51.4%) participants. Detailed baseline symptom and radiological characteristics are presented in Table 2.

**Table 2:** Symptoms and radiological findings at baseline (n = 72).

Variable	Category	Value
Pain medication use before the two-week discontinuation period	Less than once per week, n (%)	11 (15.3)
	1–2 times per week, n (%)	16 (22.2)
	Three times per week, n (%)	37 (51.4)
	Most days of the week, n (%)	8 (11.1)
Duration of pain (months)	Mean ± SD	17.3 ± 7.0
History of knee joint injury	Yes, n (%)	16 (22.2)
Kellgren-Lawrence OA grading	Grade 2, n (%)	24 (33.3)
	Grade 3, n (%)	37 (51.4)
	Grade 4, n (%)	11 (15.3)

Repeated-measures ANOVA demonstrated statistically significant changes in all clinical outcome measures across the follow-up period (all p < 0.001). Overall, WOMAC pain, WOMAC stiffness, WOMAC physical function, and VAS pain scores improved, while the 6MWD and IKDC scores increased compared with baseline. The greatest improvement in clinical outcomes was observed at the one-month follow-up, although all outcome measures remained improved at six months compared with baseline. No immediate adverse events were observed during the 24-hour post-procedure observation period. No delayed procedure-related adverse events were reported during the one-, three-, or six-month follow-up assessments. Detailed clinical outcome scores are presented in Table 3.

**Table 3:** Comparison of the clinical outcome scores at baseline and follow-up intervals (n = 72). * shows repeated measures ANOVA; p < 0.05 is considered statistically significant. SD: standard deviation; 6MWD: six-minute walking distance; VAS: Visual Analogue Scale; IKDC: International Knee Documentation Committee.

Outcome measures	Baseline mean ± SD	One-month mean ± SD	Three-month mean ± SD	Six-month mean ± SD	ANOVA (F)	p-value*
WOMAC pain (0-20)	16.3 ± 1.5	6.5 ± 1.1	8.0 ± 1.1	13.1 ± 1.4	248.61	<0.001*
WOMAC stiffness (0-8)	6.7 ± 0.8	3.5 ± 0.7	4.6 ± 0.8	5.9 ± 0.9	126.48	<0.001*
WOMAC physical function (0-68)	52.8 ± 6.1	21.1 ± 3.5	24.3 ± 3.6	27.4 ± 3.4	372.94	<0.001*
VAS pain (0-10)	7.7 ± 1.0	3.6 ± 0.8	4.3 ± 0.8	5.2 ± 0.7	198.37	<0.001*
6MWD (meters)	60.3 ± 8.3	106.8 ± 17.0	96.0 ± 14.3	86.5 ± 13.2	289.15	<0.001*
IKDC score (0-100)	45.5 ± 8.3	71.7 ± 8.6	64.4 ± 6.5	57.3 ± 5.5	241.73	<0.001*

## Discussion

In this prospective cohort study, intra-articular PRP injection was associated with significant improvements in pain and functional outcomes among patients with knee OA over a six-month follow-up period. The majority of participants were aged 51-60 years, with a slight female predominance, consistent with the known epidemiology of OA [1]. A large proportion of patients were overweight or obese, and many were engaged in physically demanding activities, both of which are established risk factors for OA progression [20-22]. Additionally, a substantial proportion of patients had advanced radiographic disease (Kellgren-Lawrence grades 3-4), which may have influenced clinical outcomes [23]. The study included participants with Kellgren-Lawrence grades 2-4, reflecting the range of disease severity encountered in clinical practice. However, the study was not specifically powered or designed to compare treatment-associated changes across individual radiographic grades. Differences in response according to radiographic severity, therefore, could not be reliably established. This heterogeneity may have contributed to variability in clinical outcomes and limits the generalizability of the findings.

Although statistically significant improvements were observed in the clinical and functional outcome measures following PRP injection, these findings should be interpreted cautiously. Statistical significance does not necessarily indicate clinical significance, and the present study did not specifically assess minimal clinically important differences. Therefore, the observed improvements should be considered as changes in patient-reported pain and function over the follow-up period rather than definitive evidence of clinically meaningful treatment effectiveness. However, an important finding of this study was that the magnitude of improvement was greatest at one month and progressively declined by six months, although outcomes remained significantly improved compared with baseline values. This trend suggests that while PRP may provide short-term symptomatic relief, its therapeutic effects may diminish over time [24]. Although the magnitude of improvement declined by six months, outcomes remained improved compared with baseline. Because repeat PRP administration was not evaluated in this study, the optimal timing or need for repeat injections cannot be determined from the present findings [24].

Our findings are broadly consistent with those of previous studies demonstrating short- to mid-term benefits of PRP in knee OA [5,6]. Ghai et al. [20] reported comparable improvements in WOMAC and VAS scores in a randomized study. Other cohort studies have reported similar findings despite variations in PRP preparation protocols. Comparative studies have also suggested that PRP may provide superior symptomatic relief compared with hyaluronic acid [10]. However, the available evidence remains inconsistent. A large randomized controlled trial reported no significant benefit of PRP compared with placebo, highlighting the ongoing uncertainty surrounding its efficacy [7,9].

The heterogeneity of findings across studies may be explained by differences in patient selection, outcome measures, follow-up duration, and PRP preparation techniques [8-11]. In the present study, PRP was prepared using a double-centrifugation technique; however, detailed characterization of platelet concentration and leukocyte content was not performed for all patients. This limitation may affect reproducibility and contribute to variability in treatment outcomes, as reported in previous studies [12,13].

OA represents a growing global health concern, particularly among aging populations. The World Health Organization has emphasized the importance of addressing age-related conditions such as OA, which substantially affect functional independence [16]. OA has also been associated with increased mortality and considerable morbidity [25]. In many low- and middle-income countries, including India, joint replacement surgery may not be readily accessible or affordable, underscoring the need for effective non-surgical treatment options [26].

Several biological mechanisms have been proposed to explain the therapeutic effects of PRP. Growth factors released from platelet alpha granules, including platelet-derived growth factor, transforming growth factor-beta, and vascular endothelial growth factor, are believed to exert anti-inflammatory effects, enhance tissue repair, and support cartilage homeostasis [23-26]. However, the clinical relevance of these mechanisms remains incompletely understood, particularly in the absence of standardized preparation methods and dosing protocols [8,9].

Strengths and limitations

The strengths of this study include its prospective design, standardized single intra-articular PRP administration, predefined clinical and functional outcome measures, and follow-up assessments extending to six months. The use of multiple complementary outcome measures, including WOMAC, VAS, IKDC, and 6MWD, allowed assessment of changes in pain, symptoms, and functional performance over time.

Several limitations should be considered when interpreting the findings. First, the study used a single-arm design without a control or comparator group; therefore, the observed improvements cannot be attributed exclusively to PRP, and placebo effects, natural fluctuations in symptoms, and regression toward the mean may have contributed to the results. Second, participants with Kellgren-Lawrence grades 2-4 were included, representing a heterogeneous range of radiographic disease severity. The study was not designed or powered to determine whether treatment response differed by individual radiographic grade. Third, although the PRP preparation protocol was operationally standardized, comprehensive characterization of the final PRP product was not available for the entire cohort. Platelet concentration was assessed in only three participants, while leukocyte concentration, platelet recovery, activation status, and relative centrifugal force were not systematically documented. Consequently, the precise biological composition of the PRP preparation cannot be fully characterized or reproduced from the available study records.

In addition, concomitant interventions during follow-up, including analgesic or NSAID use, physiotherapy, structured exercise, brace use, and weight-management interventions, were not systematically standardized. Their potential influence on the observed longitudinal changes therefore cannot be completely excluded. The study also did not specifically assess minimal clinically important differences; consequently, statistically significant changes should not be interpreted as definitive evidence of clinically meaningful benefit. Finally, the six-month follow-up period limits conclusions regarding longer-term durability of the observed improvements, and the study did not evaluate repeat PRP administration. Further controlled studies with standardized PRP characterization, longer follow-up, and appropriate assessment of clinically meaningful outcomes are warranted.

## Conclusions

Intra-articular PRP injection was associated with significant short-term improvements in pain and functional outcomes among patients with knee OA. Improvements were observed across multiple outcome measures, including the WOMAC, VAS, IKDC subjective knee score, and 6MWD, at one, three, and six months following treatment. However, the magnitude of improvement declined over time, suggesting that the therapeutic effects of PRP may diminish by six months. Given the absence of a control group and the limited follow-up duration, these findings should be interpreted with caution. Further randomized controlled trials with longer follow-up periods and standardized PRP preparation protocols are warranted to establish the long-term efficacy and optimal use of PRP in the management of knee OA.

## References

[1] GBD 2021 Osteoarthritis Collaborators (2023). Global, regional, and national burden of osteoarthritis, 1990-2020 and projections to 2050: a systematic analysis for the Global Burden of Disease Study 2021. Lancet Rheumatol.

[2] Heidari B (2011). Knee osteoarthritis prevalence, risk factors, pathogenesis and features: part I. Caspian J Intern Med.

[3] Skou ST, Roos EM, Laursen MB, Rathleff MS, Arendt-Nielsen L, Simonsen O, Rasmussen S (2015). A randomized, controlled trial of total knee replacement. N Engl J Med.

[4] Rai D, Singh J, Somashekharappa T, Singh A (2021). Platelet-rich plasma as an effective biological therapy in early-stage knee osteoarthritis: one year follow up. SICOT J.

[5] Kolasinski SL, Neogi T, Hochberg MC (2020). 2019 American College of Rheumatology/Arthritis Foundation Guideline for the management of osteoarthritis of the hand, hip, and knee. Arthritis Rheumatol.

[6] Kennedy MI, Whitney K, Evans T, LaPrade RF (2018). Platelet-rich plasma and cartilage repair. Curr Rev Musculoskelet Med.

[7] Bennell KL, Paterson KL, Metcalf BR (2021). Effect of intra-articular platelet-rich plasma vs placebo injection on pain and medial tibial cartilage volume in patients with knee osteoarthritis: the RESTORE randomized clinical trial. JAMA.

[8] Hong M, Cheng C, Sun X, Yan Y, Zhang Q, Wang W, Guo W (2021). Efficacy and safety of intra-articular platelet-rich plasma in osteoarthritis knee: a systematic review and meta-analysis. Biomed Res Int.

[9] Nie LY, Zhao K, Ruan J, Xue J (2021). Effectiveness of platelet-rich plasma in the treatment of knee osteoarthritis: a meta-analysis of randomized controlled clinical trials. Orthop J Sports Med.

[10] Aw AA, Leeu JJ, Tao X, Bin Abd Razak HR (2021). Comparing the efficacy of dual platelet-rich plasma (PRP) and hyaluronic acid (HA) therapy with PRP-alone therapy in the treatment of knee osteoarthritis: a systematic review and meta-analysis. J Exp Orthop.

[11] Sax OC, Chen Z, Mont MA, Delanois RE (2022). The efficacy of platelet-rich plasma for the treatment of knee osteoarthritis symptoms and structural changes: a systematic review and meta-analysis. J Arthroplasty.

[12] Bansal H, Leon J, Pont JL, Wilson DA, Bansal A, Agarwal D, Preoteasa I (2021). Author correction: Platelet‑rich plasma (PRP) in osteoarthritis (OA) knee: correct dose critical for long term clinical efficacy. Sci Rep.

[13] Christgau M, Moder D, Hiller KA, Dada A, Schmitz G, Schmalz G (2006). Growth factors and cytokines in autologous platelet concentrate and their correlation to periodontal regeneration outcomes. J Clin Periodontol.

[14] Kellgren JH, Lawrence JS (1957). Radiological assessment of osteo-arthrosis. Ann Rheum Dis.

[15] Bellamy N, Buchanan WW, Goldsmith CH, Campbell J, Stitt LW (1988). Validation study of WOMAC: a health status instrument for measuring clinically important patient relevant outcomes in antirheumatic drug therapy in patients with osteoarthritis of the hip or knee. J Rheumatol.

[16] World Health Organization (2000). Obesity: preventing and managing the global epidemic. report of a WHO consultation. World Health Organ Tech Rep Ser.

[17] Huskisson EC (1974). Measurement of pain. Lancet.

[18] Irrgang JJ, Anderson AF, Boland AL (2001). Development and validation of the international knee documentation committee subjective knee form. Am J Sports Med.

[19] ATS Committee on Proficiency Standards for Clinical Pulmonary Function Laboratories (2002). ATS statement: guidelines for the six-minute walk test. Am J Respir Crit Care Med.

[20] Ghai B, Gupta V, Jain A, Goel N, Chouhan D, Batra YK (2019). [Effectiveness of platelet rich plasma in pain management of osteoarthritis knee: double blind, randomized comparative study]. Braz J Anesthesiol.

[21] Nüesch E, Dieppe P, Reichenbach S, Williams S, Iff S, Jüni P (2011). All cause and disease specific mortality in patients with knee or hip osteoarthritis: population based cohort study. BMJ.

[22] Abang IE, Anisi CO, Asuquo J, Agweye P, Ngim NE, Mpama EA (2019). Osteoarthritis: knowledge and acceptability of total joint replacement. EAS J Orthop Physiother.

[23] Chen PH, Chen X, He X (2013). Platelet-derived growth factors and their receptors: structural and functional perspectives. Biochim Biophys Acta.

[24] van Buul GM, Koevoet WL, Kops N (2011). Platelet-rich plasma releasate inhibits inflammatory processes in osteoarthritic chondrocytes. Am J Sports Med.

[25] Ogino Y, Ayukawa Y, Kukita T, Koyano K (2006). The contribution of platelet-derived growth factor, transforming growth factor-beta1, and insulin-like growth factor-I in platelet-rich plasma to the proliferation of osteoblast-like cells. Oral Surg Oral Med Oral Pathol Oral Radiol Endod.

[26] Kabiri A, Esfandiari E, Esmaeili A, Hashemibeni B, Pourazar A, Mardani M (2014). Platelet-rich plasma application in chondrogenesis. Adv Biomed Res.

